# Analysis on the imbalance of population flow network during the Spring Festival travel rush in China in 2015

**DOI:** 10.1371/journal.pone.0249520

**Published:** 2021-04-05

**Authors:** Yanpeng Gao, Xiaofei Xu, Ye Wei

**Affiliations:** 1 School of Architecture, Northeastern University, Shenyang, Liaoning Province, China; 2 Grammar School, Northeastern University, Shenyang, Liaoning Province, China; 3 School of Geographical Sciences, Northeast Normal University, Changchun, Jilin Province, China; Institute of Geographic Sciences and Natural Resources Research (IGSNRR), Chinese Academy of Sciences (CAS), CHINA

## Abstract

This paper analyzes the imbalance of interprovincial population flow during the Spring Festival travel rush in China, using big data obtained through Baidu Migration, in terms of population flow during the festival and the normalized unbalanced coefficients of edge and node method for analysis, from which the following findings emerge: (1). The imbalance in population flow network during the Spring Festival travel rush is significant, with unbalanced coefficients and relevant frequencies of the population flow network in the Eastern and Western Regions being significantly higher than in other regions. The unbalanced coefficients in the Central Region are lower, followed by corresponding frequencies, while the unbalanced coefficients in the Northeast Region are evenly distributed with the lowest frequencies. The population flow toward the West and Northwest are relatively concentrated, while the population flow toward the South and Southwest are relatively scattered. (2). The regional imbalance during the Spring Festival travel rush has characteristics of spatial agglomeration, where a strongly-connected Southeast Subsystem and a weakly-connected Western Subsystem are formed; there is a significant leverage effect in Guangdong Province, which greatly affects the regional imbalance. Three characteristics emerge in the distribution of regional population flow—the outflow, inflow, and outflow along the Eastern, Central and Western strips/lines, respectively. The paper emphasizes the importance of researching imbalance issues, clarifies the difference between the imbalance of the population flow network and the imbalance involved in previous population research fields, and discusses the Spring Festival Effect in terms of population flow and deficiencies in research.

## 1. Introduction

“The major contradiction of our society has been transformed into a contradiction between the growing needs of the people for a better life, and the unbalanced and inadequate development,” which is one of the most typical features of new urbanization [1, 2] and pointed out by Xi Jinping, the president of the People’s Republic of China in the report of the 19th National Congress of the Communist Party of China. It can be seen that imbalance is one of the two main causes of the current social contradictions and also one of the main problems in the development process of China. Imbalance is considered as a classic problem in many disciplines, especially existing and being extensively studied in the field of population mobility. As early as 1990s, a number of studies on the balance and imbalance models of population flow sprang up in several countries [3–5]. But at that time, the focus was more on the economic explanations of the population flow mechanism and the economic effect in population flow in general [6, 7], which was, strictly speaking, not about either the characteristics of population flow, or the imbalance of population flow. From previous experience, the author believes that the imbalance of population flow should be defined as:

*The population flow between cities, regions, urban, and rural areas in a specific historical period varies in terms of scale, quality*, *composition, and direction, that causes the difference in population between these areas is essentially the* “*imbalance*”.

The imbalance can be considered holistically, such as by analyzing the imbalance of the nationwide population flow, or it can be spatially focused, such as measuring the imbalance between two cities. For the problem of population flow in China during the transition period, balance is a theoretical state that is hard to achieve, while imbalance is the normal state. To some extent, imbalance can enhance exchanges between regions, promote the optimal allocation of economic resources such as the labor force and become the impetus for cross-regional urbanization development [8]; however, severe imbalance can lead to regional disequilibrium, economy decline in outflow cities and the Big City Disease in inflow cities [9–11].

In the field of population mobility, “imbalance,” in a manner, similar to concepts like “vulnerability,” “coordination,” and “security,” is considered as a conceptual tool that proposes to solve certain problems. In fact, some researchers have discussed the issue of imbalance but have not clarified its scientific connotation. A review of existing literature on the subject reveals that scholars have focused mainly on the following two aspects of imbalance of China’s population flow: (1). The distribution imbalance of population flow, that is, observation of population flow from a static perspective. This perspective draws on traditional research thinking about regional imbalance by taking the city and region as the research unit and mainly uses the scale of population flow, net migration rate, total migration rate, Gini coefficient, and other indicators to describe differences in the spatial distribution of population flow. There has been abundant research in this aspect, for example, Liang et al. [12] explored the spatial mobility of interprovincial population flow based on the tables of China’s population census and the micro data samples of population census [12]; Duan [13] adopted the measurement model of population flow and economic growth to measure the regional disparity of population distribution in three regions: China as a whole, and the Eastern and the Western regions of China separately [13]; Liu et al. [15] identified the balanced active region of population flow and other types of regions by using the composite index of net migration rate and total migration rate [14]; Cao et al. [15] used the data of long tables obtained from the 5^th^ and the 6^th^ Population Census to explore population migration changes [15]; Li [18] explored population distribution from the multicenter and dispersion dimensions and accordingly identified the spatial structure of different types of cities [16]. It is not difficult to see that the static perspective pays too much attention to cities and regions, but inadequate attention to the population flow itself, so the breadth and depth of analysis is limited. (2). The correct perspective for the imbalance of population flow network is the dynamic observation of population flow. The issue of population flow cannot be set aside due to the overarching attention paid to the origin and destination. The origin and destination, the flow routes and the population flow involved, inevitably form a complex network, that is, the population flow network. In recent years, with the support of information and communication technologies, the access to population flow data has gradually become diversified, especially due to the emergence of big data, which has promoted more detailed research on the population flow network, spatial structure [17] and man-land relationship [18]. For example, Li et al. [19], Wei et al. [20], Pan et al. [21] made meaningful inroads into researching this issue by using big data such as Baidu Migration, Tencent Migration, and 360 Migration, to study the population flow network [19–21]. Compared with the static perspective of traditional spatial distribution, the imbalance of population flow network not only focuses on the node itself (traditional cities and regions), but also focuses on the interaction between nodes and the process of population flow. The measurement of imbalance is no longer based on the comparison of high and low differences, but focuses on the exchange results of “inflow and outflow” between nodes; in addition, through the comprehensive analysis of the imbalance between nodes and edges, judgments about the imbalance of the entire network system can also be made.

To summarize, based on the interprovincial population flow network during the Spring Festival travel rush in China, this paper utilizes the normalized unbalanced coefficient to measure the imbalance of population flow in China. The method eliminates the interference of the original data of inflow and outflow, making inter-provincial migration comparative. The scope of application of normalized unbalanced coefficient as a method to measure the imbalance is not just limited to the national scale, but can also be extended to the regional and global scale, as well as to the analysis of traffic network, social network, knowledge network, and other fields.

## 2. Research data and methods

### 2.1 Data

The term “Spring Festival travel rush” refers to the phenomenon where a huge number of people travel around the Spring Festival of China, which happens every year during the transition period. It first appeared in the 1980s and now the Spring Festival travel rush is known as the largest, periodic human migration in history. Due to its large scale and strong social influence, it is the focus of attention of Chinese and foreign media every year. Based on analysis, the imbalance is due to the spatial incongruity between population distribution and economic activities that then leads to the differences in the spatial distribution of population and employment; the competitiveness and attraction of the Eastern Coastal Developed Areas and big cities are much higher than those of the large swathes of developmentally backward areas in Central and Western China. Therefore, people seeking work, choose the big cities with their more developed economy and better employment opportunities. However, due to the deep-rooted traditional culture, people go home for the New Year, which results in a large number of people returning to their hometown for holidays before the Spring Festival, forming a “returning-to-hometown” flow, and after the Spring Festival, people return to their workplaces from hometowns, forming a “returning-to-workplace” flow. A huge people flow between the city and the hometown during the Spring Festival travel rush is thus generated [22]. Based on the above reasons, we decided to choose the period of Spring Festival travel as a concentrated outbreak point of population mobility and a unique window to observe the long-term population migration of China.

Research data of this paper comes from Baidu Migration, a platform launched by Baidu search engine, which provides real-time monitoring of the migration of hundreds of millions of mobile terminal users in China, based on billions of times of full sample LBS daily locator data. The emergence of this platform is a useful supplement to the widely used general survey data. For a detailed introduction of the platform and the collection and use of related data, please refer to Wei et al. [20].

This paper collected data of the population flow between 31 provinces, municipalities and autonomous regions in Mainland China (excluding Hong Kong, Macao, and Taiwan) during the Spring Festival travel rush. The officially-identified period of the Spring Festival travel rush in China is 40 days, starting from 15 days before the Spring Festival to 25 days after it. The Spring Festival travel rush in 2015 lasted from February 4^th^ to March 16^th^. Due to data acquisition, the research period of this paper is from February 7^th^ to March 18^th^, totaling 40 days (hereinafter referred to as Spring Festival). The basic data is the number of daily come-and-go person-time between 31 provinces (cities) during the Spring Festival; the data is structured as 40 directional weighted 31×31 matrices.

$$j_{1}\;j_{2}\;\cdots \;j_{n-1}\;j_{n}$$

$$R=\begin{array}{c} i_{1}\\ i_{2}\\ \vdots \\ i_{n-1}\\ i_{n} \end{array}\left[\begin{array}{ccccc} 0 & R_{12} & \cdots & R_{1(n-1)} & R_{1n}\\ R_{21} & 0 & \cdots & R_{2(n-1)} & R_{2n}\\ \vdots & \vdots & \vdots & \vdots & \vdots \\ R_{(n-1)1} & R_{(n-1)2} & \cdots & 0 & R_{(n-1)n}\\ R_{n1} & R_{n2} & \cdots & R_{n(n-1)} & 0 \end{array}\right]$$

**Fig 1:**
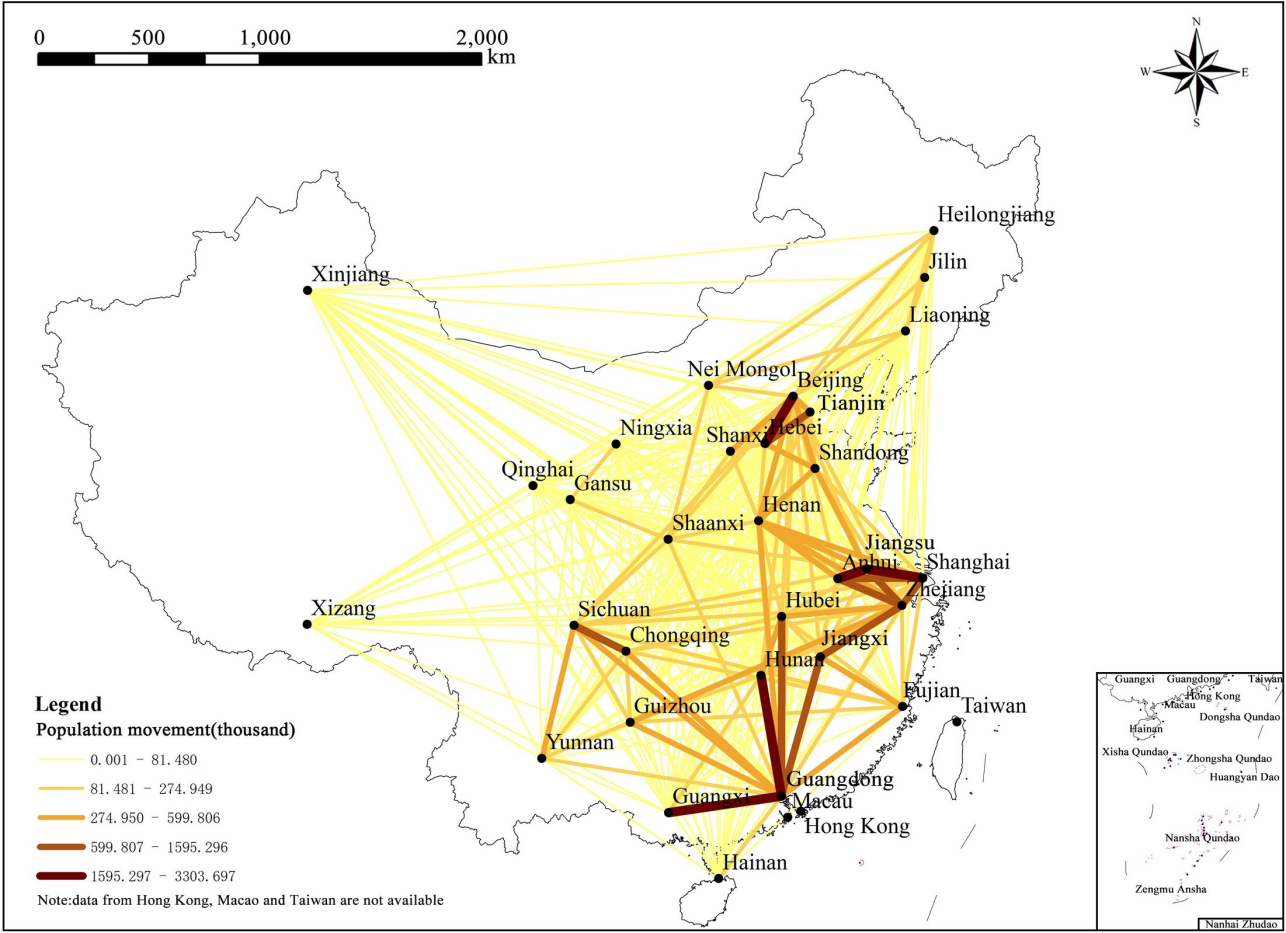
Interprovincial population flow network during the Spring Festival travel rush in China. (Image Source: https://www.naturalearthdata.com/downloads/10m-cultural-vectors/).

**Fig 2:**
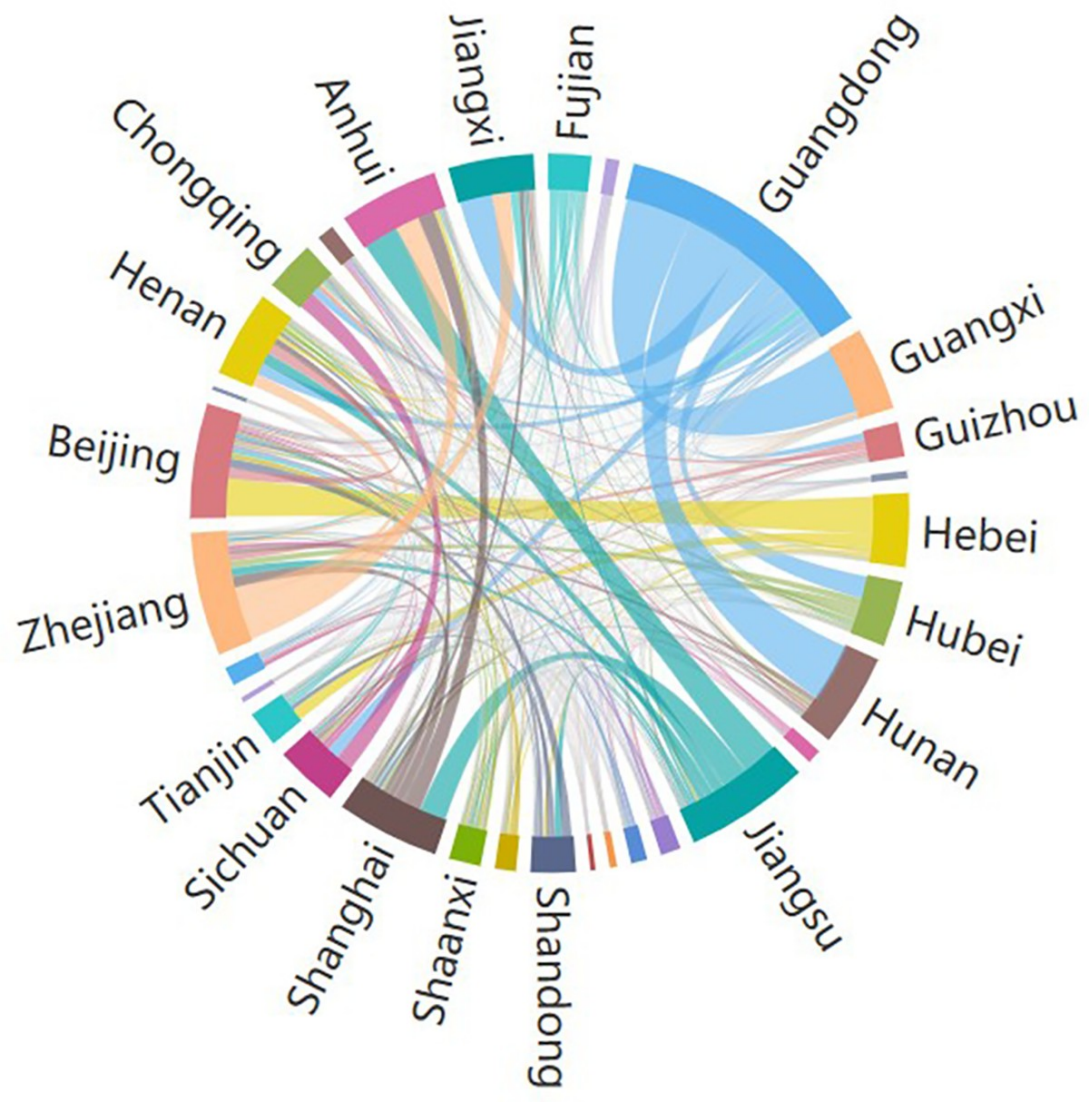
Mobility of interprovincial population flow during the Spring Festival travel rush in China.

Based on the overall observation of data acquired through Baidu Migration, it is found that China’s official holidays of Spring Festival have a significant impact on the direction of population flow. In 2015, there were 7 days for the Chinese Spring Festival national holiday, starting from February 18^th^ (New Year’s Eve of Chinese lunar year) to February 24^th^ (6^th^ day of Chinese lunar year) and February 25^th^ was the first working day. Before February 23^rd^, it was mainly the returning-to-hometown flow and after February 23^rd^, the returning-to-workplace flow would reach the peak. For two provinces (municipalities/autonomous regions), with this date as the cutting-point, the population flows before and after this point are highly symmetrical (R^2^ = 0.914, P = 0.01). Therefore, it is agreed here that the period from February 7^th^ to February 23^rd^ is considered as “Before Festival” and the period from February 24^th^ to March 18^th^ is considered as “After Festival”. In view of the fact that the population flow during the Spring Festival is generally a symmetrical round-trip process, in order to reduce data redundancy and eliminate the information interference due to data symmetry, the analysis of this paper will only focus on the Before Festival data. Figs 1 and 2 depict the population flow network and the mobility of population flow generated based on the population flow data before the festival, respectively.

It can be seen from the above figures that the population flow in each province during the Spring Festival travel rush is extremely uneven, including for Guangdong, Jiangsu, Zhejiang, and other provinces with large population flow, as well as Hainan, Ningxia, Qinghai, and other provinces with small migration scale. The migration scale is related to the population base of the province, as well as factors such **as** the spatial location, economic level, and regional development of the province. In addition, between adjacent provinces, the population flow is relatively large, such as Guangdong and Guangxi, Beijing and Hebei, and Shanghai, Jiangsu, and Anhui.

### 2.2 Methodology

To explore the imbalance of population flow network, it is necessary to not only rely on a conceptual framework, but to also use a detailed methodology. Therefore, this paper proposes the normalized unbalanced coefficient method to analyze the characteristics of the big data of Spring Festival travel rush provided by Baidu Migration and the structural characteristics of the population flow network. According to different analysis objects, the coefficients can be divided into two types:

#### 2.2.1 Normalized unbalanced coefficients of edge

To study the imbalance of the connection between two provinces (municipalities/autonomous regions), it is not enough to only measure the net flow, but is also necessary to refer to the overall scale of two-way flow between the two provinces (municipalities/autonomous regions). For example, suppose the net flow between two provinces (municipalities/autonomous regions) is only 10 and the total two-way flow between the two provinces (municipalities/autonomous regions) is also 10, it indicates that the population flow between the two provinces (municipalities/autonomous regions) is leaning toward one side and strongly-unbalanced. If however, with the same net flow 10 and a total two-way flow of 10,000 between the two provinces (municipalities/autonomous regions), it can be seen that the net flow is obviously insignificant compared with the total two-way flow, then the flow between the two provinces (municipalities/autonomous regions) is in a balanced state. Based on the above considerations, a normalized unbalanced coefficient is proposed in this paper and its calculation formula is as follows:

$${NUB}_{ij}=\frac{R_{ij}-R_{ji}}{R_{ij}+R_{ji}},\;i\neq j\;and\;R_{ij}>R_{ji}$$ (1)

Where, *NUB*_*ij*_ is the normalized unbalanced coefficient of population flow between province *i* (municipalities/autonomous regions) and province *j* (municipalities/autonomous regions), *R*_*ij*_ represents the population flow from province *i* (municipalities/autonomous regions) to province *j* (municipalities/autonomous regions) before and after the Spring Festival, and *R*_*ji*_ represents the population flow from province *j* (municipalities/autonomous regions) to province *i* (municipalities/autonomous regions) before and after the Spring Festival. The numerical value of *NUB*_*ij*_ is between 0 and 1, and the purpose of normalization is to reduce the influence of absolute amount on the judgment of the calculated result. The essence of *NUB*_*ij*_ is to describe the proportion of two-way net flow to the total two-way flow. If the value is close to 1, it indicates that one-way flow is significant and equilibrium is low; if the value is close to 0, it indicates that two-way flow is significant and equilibrium is high. Based on the above formula, since the normalized unbalanced coefficients of edge of the two provinces with population flows are a pair of opposite numbers, then *NUB*_*ij*_ is recorded as the calculated value under the condition of *R*_*ij*_>*R*_*ji*_.

#### 2.2.2 Normalized unbalanced coefficient of node

The calculation method of the normalized unbalanced coefficient of node is similar to that of the edge; the calculation formula is as follows:

$${NUB}_{i}=\frac{\sum \left(R_{ij}-R_{ji}\right)}{\sum \left(R_{ij}+R_{ji}\right)},\;i\neq j$$ (2)

Where, *NUB*_*i*_ represents the normalized unbalanced coefficient of node; the other variables are the same as above. It is obvious that the normalized unbalanced coefficient of node is between [–1, 1]. When the node is in the net outflow state, then *NUB*_*i*_∈(0, 1]; when the node is in the net inflow state, then *NUB*_*i*_∈[–1,0]; when the outflow equals the inflow, then *NUB*_*i*_ = 0. When the absolute value of *NUB*_*i*_ is greater than 0.5, it indicates that the imbalance is high, otherwise, the imbalance is low.

## 3. Result analysis

### 3.1 Analysis of unbalanced coefficients based on edges

#### 3.1.1 Frequencies of population flow network

**Fig 3:**
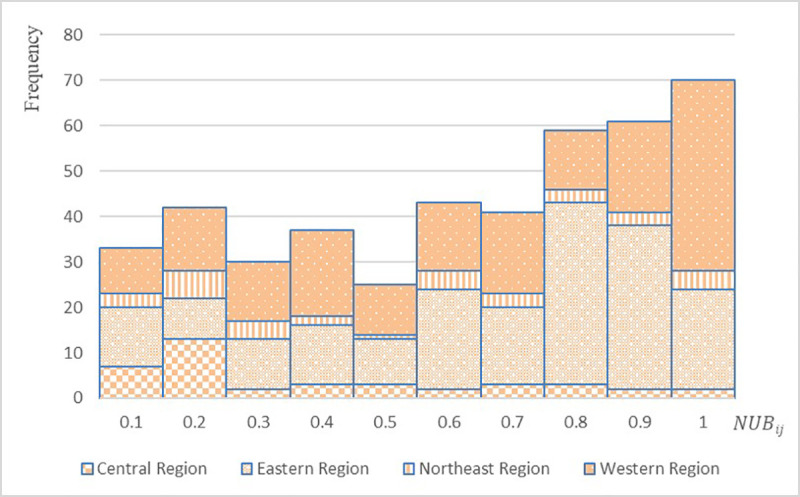
Frequency distribution histogram of the unbalanced coefficients of population flow network during the Spring Festival travel rush in China.

According to Formula (1), we calculate the unbalanced coefficient of population flow network of each province (municipality/autonomous region) during the Spring Festival travel rush in China, classify the departure places of population flow based on the four major economic regions of China, calculate the statistics on the occurrence frequency of the unbalanced coefficient and get the frequency distribution histogram (Fig 3).

As shown in Fig 3, it is confirmed that imbalance exists in the population flow network during the Spring Festival travel rush in China and an obvious peak value is visible on the histogram around 0.7~1, which generally presents a trend leaning toward the right side of the histogram, which indicates that the imbalance of population flow network during the Spring Festival travel rush in China is relatively high. According to the statistical results based on economic regions, the unbalanced coefficient and frequency of the population flow network with departure places in the Eastern Region and Western Region are significantly higher than those in other regions, which proves that the population attraction of the above regions is relatively high and the scale of population flow is relatively large, with a wide range of population flow destinations. The unbalanced coefficient of the population flow network in the Central Region is generally low and the frequency of unbalanced coefficient is secondary to it, which indicates that the population flow in the Central Region is relatively balanced. The distribution of the unbalanced coefficient of population flow network in Northeast China is relatively average with the lowest frequency of unbalanced coefficient, indicating that the destinations of the population flow are limited to only a few places and the imbalance of different destinations varies greatly.

#### 3.1.2 Directionality of population flow network

**Fig 4:**
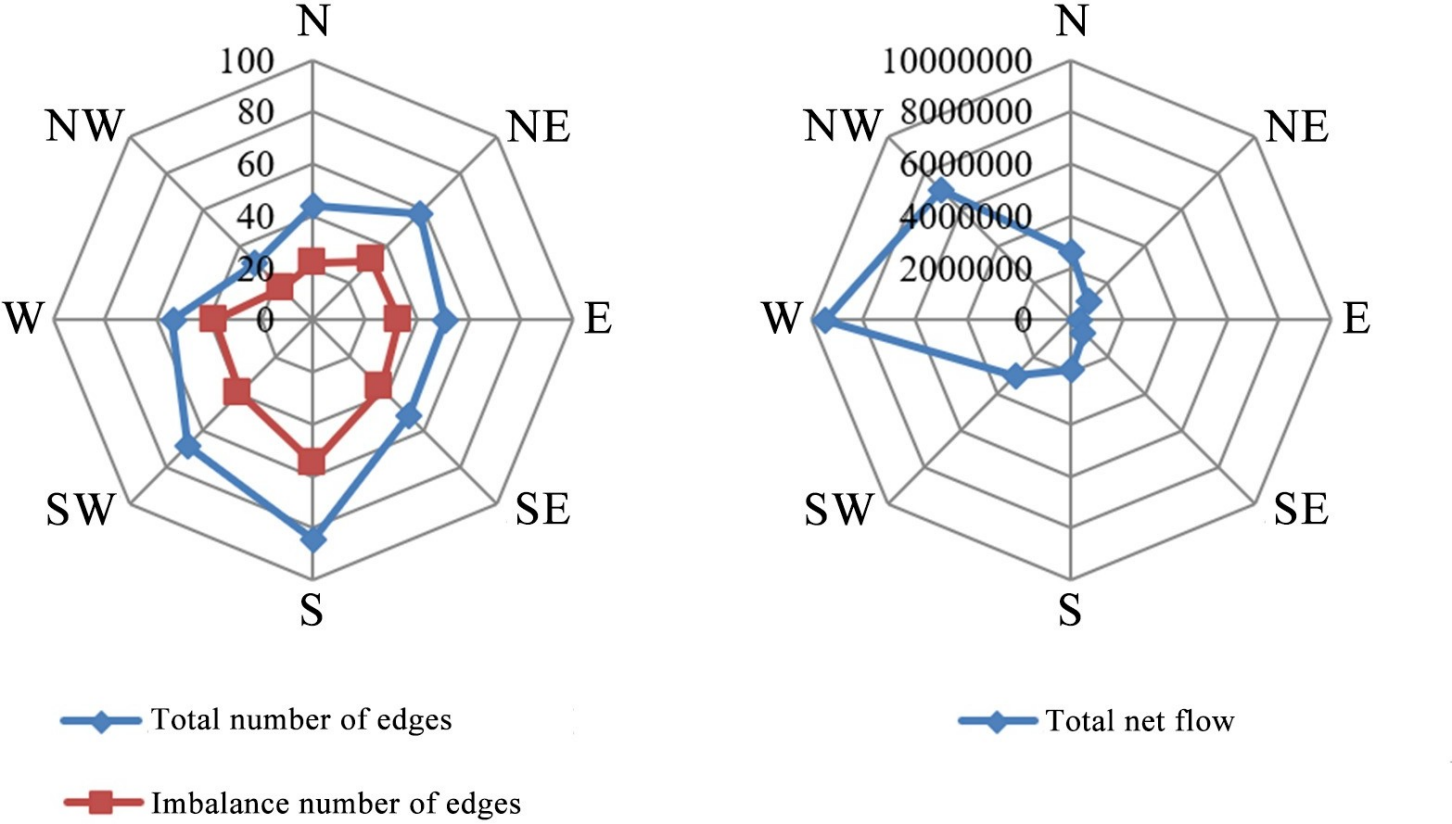
The directional characteristics of population flow during the Spring Festival travel rush in China.

Given that the imbalance coefficient of edge only represents the level of imbalance between two places with population flow, it cannot reflect the direction of population flow. In order to analyze in more detail, we use ArcGIS software to calculate the azimuth of population flow and attribute these angles of azimuth to eight quadrants of directions—East, Northeast, North, Northwest, West, Southwest, South, and Southeast, so as to generate statistics on the total number of edges, unbalanced edges, and total net flow contained in each quadrant of direction, which form a radar map, as shown in Fig 4.

From Fig 4(A), it can be seen that during the Spring Festival travel rush, the total number of edges of southward population flow is the highest, followed by the population flow toward the Southwest, which is then followed by the population flow toward the Northeast, while the lowest population flow appears toward the Northwest; the distribution in other directions is relatively even. From Fig 4(B), it can be seen that during the Spring Festival travel rush, there is a significant difference in the direction of the total net flow. Directions with the largest value of total net flow are the West and the Northwest and a notable difference is noticeable in other directions. Before the Spring Festival, the population flow is characterized with a significant imbalance from East to West. Though the number of edges to the West and Northwest are very few, the net flow of population is highly concentrated, which is the main cause for the imbalance of the total net flow in different directions.

According to the results of Fig 4(A) and 4(B), during the Spring Festival travel rush, although there are a small number of unbalanced edges toward the West and Northwest, the total net flow is quite big; hence, the population flow of this direction is relatively concentrated, corresponding to the population flow of “Central Region→Western Region” and “Eastern Region→Central Region”. On the contrary, the population flows toward South and Southwest are relatively scattered.

### 3.2 Analysis of unbalanced coefficients based on nodes

**Fig 5:**
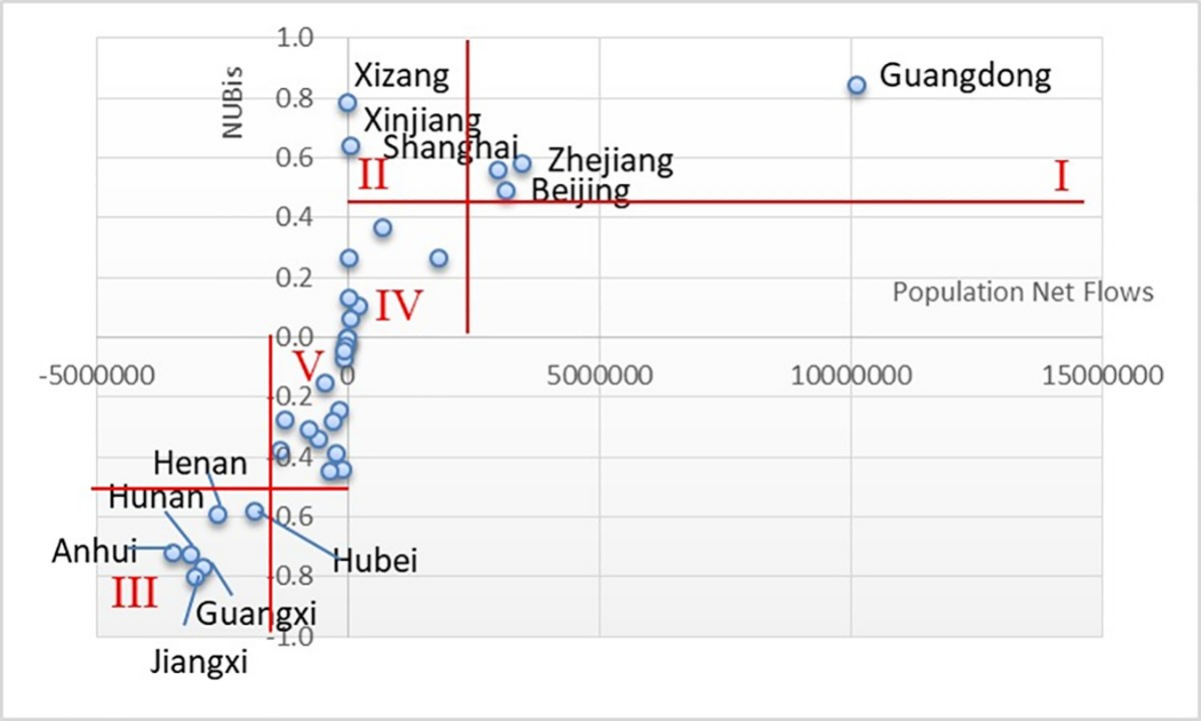
Scatter map of population net flow and normalized unbalanced coefficient (*NUB*_*i*_).

In existing literature, the net flow is often used as an index to measure the imbalance between the inflow and outflow of nodes. Therefore, in order to take into account the imbalance and directionality, this paper draws the scatter map of cities by taking the imbalance coefficient *NUB*_*i*_ as the vertical coordinate and the net flow as the horizontal coordinate (Fig 5). According to the distribution characteristics of the scatter map, provinces (municipalities/autonomous regions) of China can be classified into five types:

- Type I—high-imbalance & high-outflow type, which consists of four regions: Guangdong, Zhejiang, Shanghai, and Beijing, corresponding to the three most developed metropolitan areas in China and have a dominant position in the population flow network.
- Type II—high-imbalance & low-outflow type, which consists of two regions: Xizang and Xinjiang. Although the two regions do not have a large amount of population flow, they are still in the state of net outflow, with a high level of imbalance, which are the special characteristics of these regions.
- Type III—high-imbalance & high-inflow type, which consists of six regions: Anhui, Henan, Hubei, Hunan, Jiangxi, and Guangxi.
- Type IV—low-imbalance & low-outflow type, which consists of six regions: Liaoning, Tianjing, Jiangsu, Fujian, Ningxia, and Qinghai.
- Type V—low-imbalance & low-inflow type, which consists of the largest number of provinces (municipalities/autonomous regions), including 13 regions: Heilongjiang, Jilin, Nei Mongol, Hebei, Shandong, Shanxi, Shaanxi, Gansu, Chongqing, Sichuan, Guizhou, Yunnan, and Hainan.

**Fig 6:**
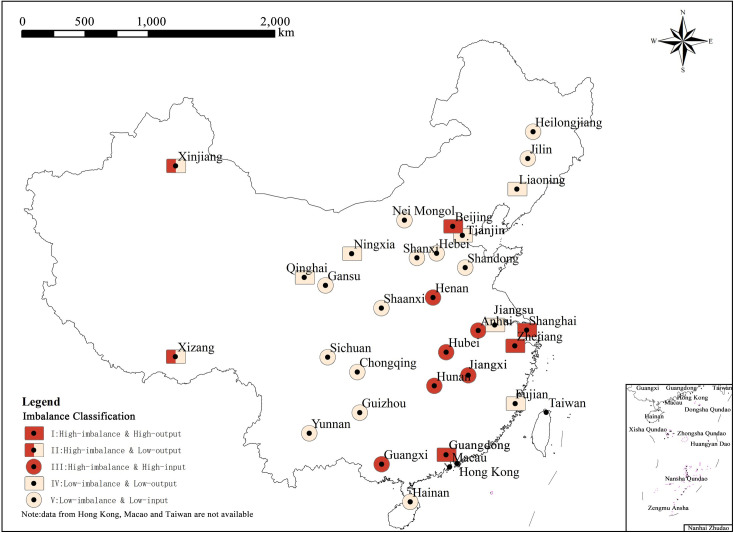
Classification of provinces (municipalities/autonomous regions) in terms of regional imbalance. (Image Source: https://www.naturalearthdata.com/downloads/10m-cultural-vectors/).

Due to the lack of data, Taiwan, Hong Kong, and Macao are not classified. In order to provide a clear and graphical explanation and analysis, spatial visualization has been done for the classification of imbalance in Fig 6, so as to summarize the main characteristics of regional imbalance during the Spring Festival travel rush.

#### 3.2.1 Spatial agglomeration

Based on the characteristics of spatial agglomeration, three distinct regions are identifiable—the Eastern Coastal Area, the Central Area, and the Western Area, which can be visualized in terms of high and low population migration in the state of high imbalance. The detailed analysis is as follows.

**Fig 7:**
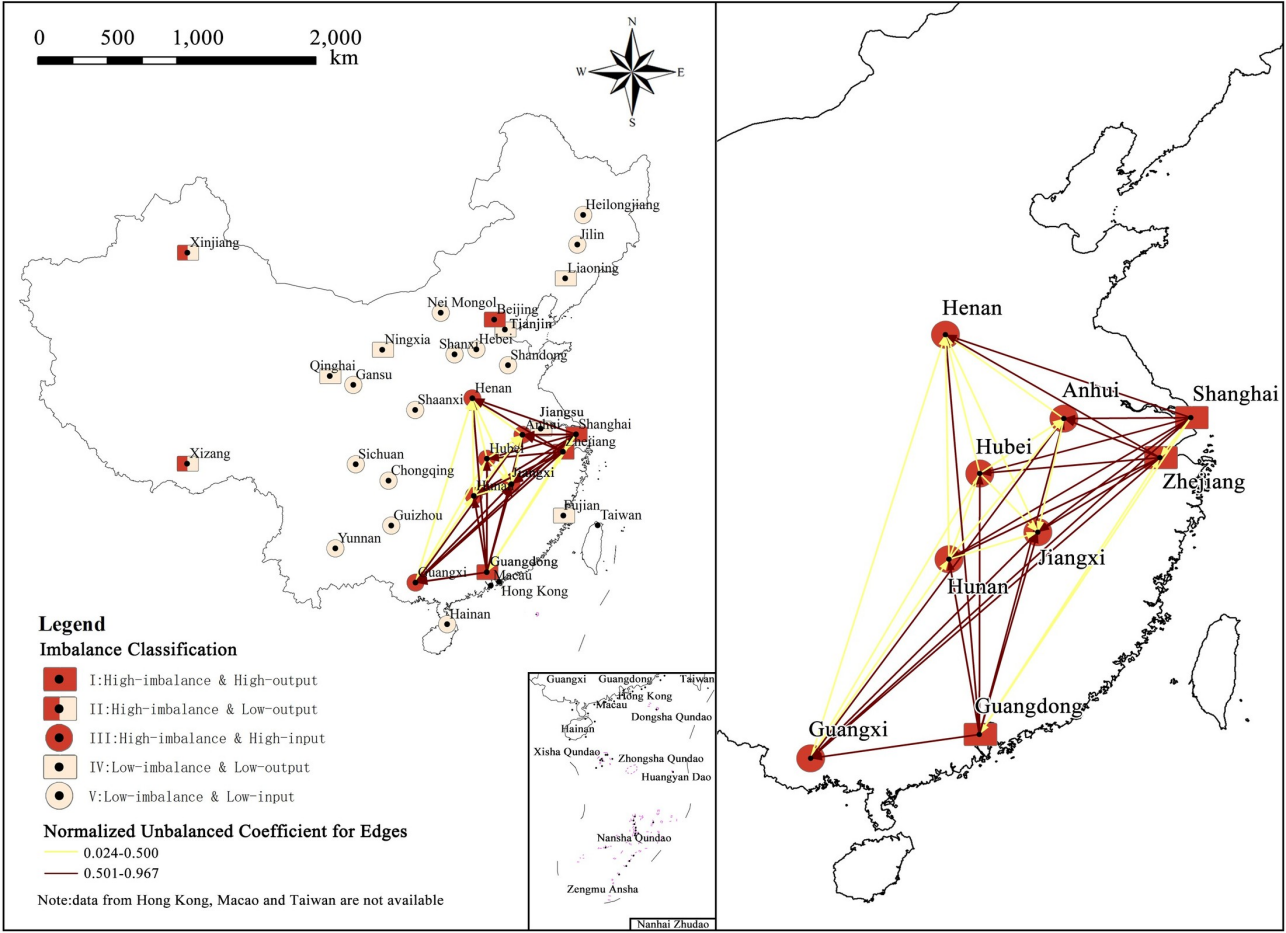
Spatial map of population flow network and regional imbalance in the Southeast Subsystem. (Image Source: https://www.naturalearthdata.com/downloads/10m-cultural-vectors/).

Type I and Type III spatial agglomeration with high-imbalance and large scale of net population flow are formed in the Eastern Coastal and Central Areas, and the spatial agglomeration features are most prominent in Type III areas. As shown in Fig 6, Type III areas are mainly in provinces of Central China, where provinces are tightly connected and form a large plate. It is clear that the regions bordering Type I areas such as Anhui, Jiangxi, Hunan, and Guangxi, have higher imbalance coefficient, and the regions not bordering Type I areas, such as Henan and Hubei, have relatively lower imbalance coefficient. The net flow shows the same pattern as well. The fact that there is a high level of imbalance in both Type III and Type I areas and the net flow directions of these areas are complementary, reveals that there is a very strong interaction and significant correlation between Type III and Type I areas. Therefore, some Type III and Type I areas form a small regional system, which is tentatively called Southeast Subsystem according to its spatial location. The spatial map of population flow and regional imbalance in the Southeast Subsystem is drawn (Fig 7) by extracting the system and coinciding with the imbalance coefficients of edges and classification of regional imbalance.

It can be seen from Fig 7 that, in addition to the population flow routes of Guangxi to Anhui and Guangxi to Jiangxi, the imbalance of population flow network among the internal nodes of Type I and Type III regions is relatively low, while there is a high level of imbalance among interval nodes. That is to say, the Southeast Subsystem shows obvious differentiation in spatial distribution: the regional imbalance is strong and the internal imbalance between regions is weak, the population flow is relatively balanced between regions of the same level, and the population flows are unbalanced between cross-level regions. The Southeast Subsystem also shows high intensity of imbalance due to its large-scale population flow.

The unbalanced agglomeration in the Western Region exists as a special case. According to basic data, the largest dominant flow of Xizang is mainly to Sichuan (net flow: 6,081), Chongqing (net flow: 930) and Qinghai (net flow: 783), while the population flow of Xinjiang is mainly to Gansu (net flow: 37,434), Henan (net flow: 6,655), Sichuan (net flow: 5,638), Shaanxi (net flow: 4,964), and Chongqing (net flow: 3,441). The imbalance coefficients of Xizang and Xinjiang are mainly affected by other regions in the West. Another small regional system is formed centered on Xizang and Xinjiang, which can be called the Western Subsystem. Compared with the Southeast Subsystem, the Western Subsystem can be classified as a strong-connection system due to its large-scale of population flow. Although the Southeast Subsystem has a higher level of imbalance, its net flow is small, so it has a less obvious impact on the mobility network nodes and shows a low level of imbalance, which is classified as a weak-connection system.

#### 3.2.2 Leverage effect

It can be seen from Fig 7 that if we only consider the number of unbalanced edges, then the unbalanced coefficient of nodes should be presented as Shanghai>Guangdong>Zhejiang. However, the actual imbalance of nodes in Guangdong (0.845) is significantly higher than that in Shanghai (0.561) and Zhejiang (0.583), which shows that in addition to the number of unbalanced edges, the regional net flow also has a significant effect on the regional imbalance. At the same time, combined with the results and scatter map of the above analysis, the imbalance coefficient and net flow are generally in a linear connection, which means that areas with bigger net flow tend to be more unbalanced, while areas with smaller net flow tend to be more balanced. Although there are only few areas with big net flow, they play a dominant role in the process that causes the imbalance and present a noticeable leverage effect.

**Fig 8:**
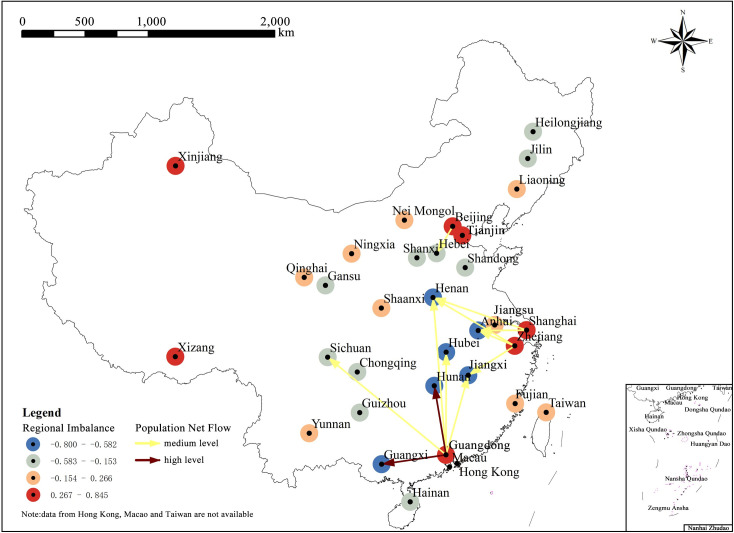
Spatial map of regional imbalance and population net flow. (Image Source: https://www.naturalearthdata.com/downloads/10m-cultural-vectors/).

To test this conjecture, this paper uses the natural breakpoint method to distinguish high, medium, and low levels of net population flow and overlaps it with regional imbalance (Fig 8). The results show that the medium and high level of net flow only exist in Beijing, Shanghai, Zhejiang, and Guangdong provinces, all of which (except for Beijing) are distributed in the southeast coastal area with high node imbalance coefficient. Among them, under the condition of substantial net flow, the population flow line only exists in Guangdong Province: during the Spring Festival travel rush in 2015, the amount of population outflow in Guangdong Province reached 11.053 million, the amount of population inflow was only 929,000 and the proportion of outflow population was 1189.8%, showing a significant imbalance. The net outflow of 10.124 million people was 1.56 times the total net flow of Shanghai and Zhejiang combined, far exceeding the migration data of Guangdong in the *6*^*th*^ *Nationwide Population Census*. All the above results confirm that the regional imbalance has a significant leverage effect in Guangdong Province.

Although nodes with leverage effect can provide large development space and a lot of opportunities for the surrounding areas, they have great significance in terms of leveraging regional development and are an important means to drive the growth of surrounding areas by promoting development of the node. However, the existence of leverage effect, on the one hand, leads to potential risks arising in network security due to excessive dependence on strong nodes; on the other hand, it also easily leads to the occurrence of local imbalances, resulting in the external effect wherein the population development opportunities of some weak nodes are constantly weakened. Therefore, in the actual development process, it is necessary to tightly control the nodes that have shown preliminary leverage effect, to prevent the strong nodes from over-development.

#### 3.2.3 Zonality

As shown in Fig 6, during the Spring Festival travel rush, the outflow areas are mainly distributed in the coastal areas and western border areas, while more provinces (municipalities/autonomous regions) in the Central Region are classified into the inflow area, which generally shows a strip/line-shaped layout along the Eastern, Central, and Western parts. For several inflow areas, the differentiation of the strip/line is also quite noticeable. The imbalance coefficient is higher in the South and the East and lower in the North and the West. No matter from the direction of inflow/outflow or the imbalance coefficient, the difference between the East and the West is obviously greater than that between the North and the South, which is mutually confirmed by the information presented in the radar map in Fig 4. Compared with outflow regions, inflow regions cover a bigger area and involve more provinces (municipalities/autonomous regions), while fewer provinces with smaller areas attract more population, presenting the characteristics of population agglomeration. Except Hebei, Shandong, Guangxi, and Hainan, the coastal areas are all classified into the outflow area, which can be attributed to the dense population of Hebei and Shandong, the weak economic level of Guangxi and Hainan, and the difficulty of population migration due to the proximity to highly-developed areas, which results in the net population outflow.

**Fig 9:**
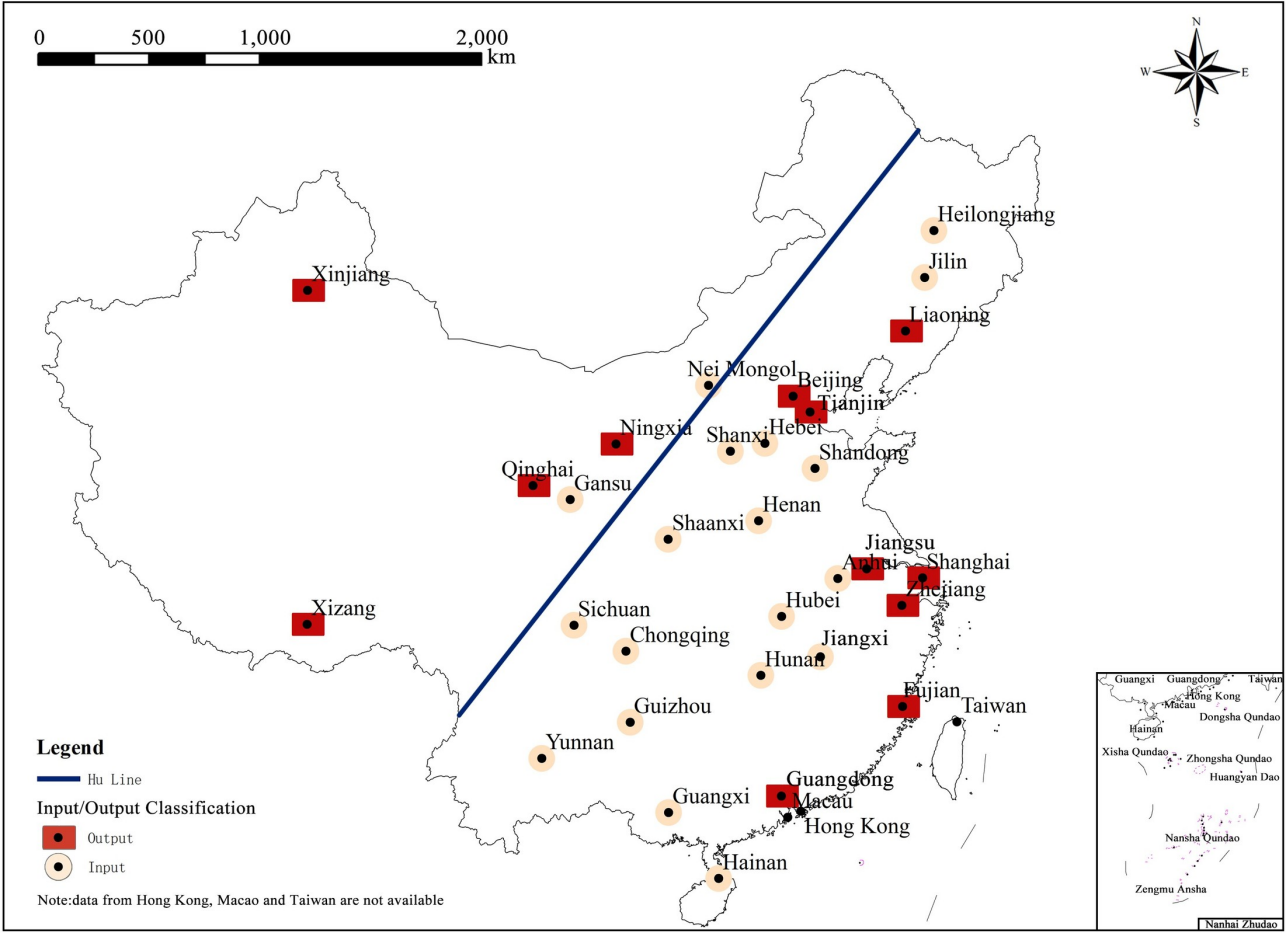
Types of population migration and the Hu Line. (Image Source: https://www.naturalearthdata.com/downloads/10m-cultural-vectors/).

In addition, by classifying provinces (municipalities/autonomous regions) according to the types of population migration, it is found that the layout of population outflow and inflow areas during the Spring Festival travel rush generally presents a strip/line spatial form that is parallel to the Hu Line (Heihe-Tengchong Line or Aihui-Tengchong Line) (as shown in Fig 9). The Hu Line is an important geographical demarcation line of China [23]. The distribution rules of cultural and geographical elements such as population density, ecological environment and economic development on both sides are quite different, so it is considered as the line dividing the population migration. The law of population migration in China has gradually changed from the West to East and from inland-to-coastal agglomeration [24] in the early stage, to the “Against the Regular Line” trend, wherein the population in the Central Region is divided by the Hu Line into the East and the West (as shown in Fig 9), which can be regarded as a signal that the regional pattern of population migration in China has started to weaken.

#### 3.2.4 Other characteristics

In addition to the above characteristics, some other details should also be paid attention to:

Type I areas such as Guangdong, Shanghai, and Zhejiang form a tightly connected combination with Type III areas in Southeast China. However, Beijing, also a Type I area, has not formed strong interaction with surrounding areas, which indicates that Beijing does not playing a significant leading role in terms of population flow with respect to its surrounding areas. However, it is not just Beijing, in North China, not a single node has created such an influence on its surrounding areas. As a province with a large population and crossing the demarcation line between the North and South, Henan Province is obviously more attracted to the southern provinces and has integrated into the population agglomeration dominated by the Yangtze River Delta and the Pearl River Delta.

Though Guangdong, together with Shanghai, Zhejiang, and Beijing, is classified into the Type I area, the net flow and the imbalance coefficients of Guangdong are much higher than those of the other three areas, which indicates that Guangdong Province has an absolute advantage in attracting the migrant population in China.

## 4. Conclusion and discussion

### 4.1 Conclusion

Based on big data on population flow during the Spring Festival travel rush obtained from Baidu Migration, this paper proposes a normalized unbalanced coefficient-based analysis of edge and node to examine the imbalance of population flow between provinces in China, and draws the following conclusions:

First, the population flow network during the Spring Festival travel rush in China presents a high level of imbalance in general. Whether measured by the imbalance coefficient of edge or node, the population flow network during the Spring Festival travel rush shows a significant level of imbalance. There are great differences in the number of sides and net flow of population in different directions, forming the characteristics of unbalanced population flow. This imbalance not only reflects the unbalanced regional development, but also reflects the unstable state of the nationwide labor market. It is speculated that this state will at least last throughout the entire rapid urbanization development process.

Second, the imbalance of population flow network during the Spring Festival travel rush in China has three characteristics: spatial agglomeration, leverage effect, and zonality. Spatial agglomeration is mainly reflected in the close connection between the highly-unbalanced outflow area and its surrounding areas, which results in the formation of a local subsystem; leverage effect is mainly reflected in the fact that some strong nodes of population flow in the network are also highly-unbalanced, and these few strong nodes play a dominant role in the network, being the main force causing the imbalance; zonality is mainly reflected in the gradient change of the imbalance in the east–west direction.

### 4.2 Discussion

First, the issue of imbalance has not attracted enough attention in the field of population mobility. Imbalance is an important index reflecting the characteristics of population flow network structure, and an important starting point to reveal the spatial benefits of population flow and to formulate a regional cooperation strategy. Though some relevant research have already been conducted, these have mainly focused on population mobility and migration rate. So far, there have not been many studies that have focused on the imbalance as a scientific problem and studied the imbalance itself, which needs to continue to be the point of emphasis in the future.

Second, imbalance research based on the population flow network proposed and attempted in this paper is obviously different from traditional imbalance research. Traditional imbalance research studies are often based on analysis of static data, and the measurement of imbalance level mainly depends on the comparison of data; whereas, the imbalance of population flow has significant vector characteristics, which not only highlights the difference in the amount of population flow, but also the difference in directionality, and places more emphasis on the dynamic interaction or competition process. The change in focus of research thinking on this issue will help open new avenues of research and enrich the research on imbalance.

Furthermore, the Spring Festival Effect of population flow. From the above analysis, it can be seen that during the Spring Festival travel rush, before the festival, the population often flows from developed areas to underdeveloped areas, while after the festival, the population flows from underdeveloped areas to developed areas, which can better reflect the laws of economics, is highly consistent with the long-term laws of population migration and provides good mapping of population migration in China during the transition period. Therefore, the significance of research on the population migration network during the Spring Festival travel rush is not only the research itself, but is also an important data source and data window to analyze the population migration issues in China.

Finally, there are some deficiencies in the research. Although the data from Baidu Migration are obtained from hundreds of millions of samples, these data only come from mobile network terminals, therefore, many people who do not have mobile terminals or do not use Baidu spatial positioning products are not included in the samples, which is a defect in the data. In addition, the data can only show the population migration process, however, the purpose of migration remains unknown. Due to the length limitation of this paper, we have not provided an in-depth discussion on the dynamic mechanism that drives the imbalance of population flow network during the Spring Festival travel rush in China, which will be supplemented in the future.

## Supporting information

S1 Data (XLS)
